# Understanding the Dissolution of Cellulose and Silk Fibroin in 1-ethyl-3-methylimidazolium Acetate and Dimethyl Sulphoxide for Application in Hybrid Films

**DOI:** 10.3390/ma17215262

**Published:** 2024-10-29

**Authors:** James A. King, Peter J. Hine, Daniel L. Baker, Michael E. Ries

**Affiliations:** School of Physics and Astronomy, University of Leeds, Leeds LS2 9JT, UK; mmjki@leeds.ac.uk (J.A.K.); p.j.hine@leeds.ac.uk (P.J.H.); d.l.baker@leeds.ac.uk (D.L.B.)

**Keywords:** silk fibroin, cellulose, composite, ionic liquid, biomaterial, biocomposite, dissolution, EmimAc, DMSO, blend

## Abstract

This paper investigates the dissolution of two biopolymers, cellulose and silk fibroin, in a mixture of 1-ethyl-3-methylimidazolium acetate (EmimAc) and dimethyl sulphoxide (DMSO). EmimAc is a promising environmentally friendly solvent currently in wide use but can be limited by its high viscosity, which inhibits the speed of dissolution. To mediate this, DMSO has been used as a cosolvent and has been shown to significantly lower the solution viscosity and aid mass transport. Dissolution experiments are carried out separately for both cellulose and silk fibrion with a range of EmimAc:DMSO ratios from 100 wt% EmimAc to 100 wt% DMSO. Interestingly, the optimal EmimAc:DMSO ratio (in terms of dissolution speed) is found to be very different for the two biopolymers. For cellulose, a mixture of 20 wt% EmimAc with 80 wt% DMSO is found to have the fastest dissolution speed, while for silk fibroin, a ratio of 80 wt% EmimAc with 20 wt% DMSO proves the fastest. These dissolution trials are complemented by rheological and nuclear magnetic resonance experiments to provide further insight into the underlying mechanisms. Finally, we produce hybrid biopolymer films from a solution to show how this work provides a foundation for future effective dissolution and the preparation of hybrid biopolymer films and hybrid biocomposites.

## 1. Introduction

Petrochemical plastic use has burdened the environment and provides an impetus for the research of sustainable and biodegradable alternatives [1]. Biopolymeric materials show particular promise and offer sustainable solutions in the medical, structural, and aerospace fields [2,3,4,5,6,7,8]. However, shortcomings in strength, hydrophobicity, and durability ultimately limit their impact [5]. Hybrid biopolymer composites, composed of multiple different polymers, can improve on material properties compared to non-hybrid alternatives [2,9,10,11,12,13,14,15,16]. In particular, blends of silk fibroin (SF) and cellulose offer unique compatibility, blending at the molecular level, and show improved properties in excess of other examples [5,17,18,19,20,21]. It is reported that hybrid composites of SF and cellulose show improved strength, biocompatability, and toughness [5,17,18,19,20,21], and retain carbon neutrality and biodegradability [22]. For example, most cellulosic materials will lose 50% of their mass within 30 days in any natural water and break down into environmentally friendly chemicals [23]. To utilise these biopolymers in large-scale applications, it is essential to understand their behaviours intimately throughout the preparation process [24].

Silk is a fibrous protein extrusion, formed of a hierarchical structure with varied chemical compositions. Silk is formed of silk sericin and SF proteins. SF, the structural protein, commonly has a hexapeptide primary sequence of mostly glycine amino acid units [25]. Raw silks are remarkably tough, flexible, and strong but can contain inherent flaws [26,27]. Existing voids allow degradation by allowing wetting and acting as water channels. Structural hydrogen bonds in biomaterials can then be broken by water molecules [27]. These shortcomings can be overcome through their inclusion in hybrid composites [5,17].

Cellulose is an anisotropic, abundant, biocompatible polymer. It is formed of repeat units of glucose [28], with a polymer chain formed of β-1,4-glycosidic bonds between two anhydroglucose monomer units [28]. Glucose monomer units are present as D-glucopyranose, the lowest energy ring conformation [5,29]. Cellulose is sparingly soluble due to extensive inter- and intramolecular hydrogen bonding and amphiphilic behaviour due to apolar hydrophobic stacking [30].

Cellulose, similarly to silk, forms complex and varied hierarchical structures at differing length scales. In nanocellulose (1–1000 nm) alone, three divisions are typically seen: cellulose nanocrystals (up to 90% crystalline particles); cellulose nanofibrils (long entangled fibrils with amorphous and crystalline phases); and bacterial cellulose (high-purity ribbon-like fibres in a web) [29]. Biopolymer solubility and behaviour is dependent on the structure, degree of polymerisation (DP), impurities, and temperature [31,32]. Typically, the source affects the molecular weight of the given polymer and thus impacts solubility. Unprocessed cellulose can have a molecular weight of more than 500,000 g mol^−1^, and standard microcystalline cellulose (MCC) has an approximate molecular weight of 29,000–36,000 g mol^−1^ [33].

Cellulose solubility has been studied more extensively than that of SF and shows the importance of researching improved solvation techniques. Historically, due to the insoluble nature of cellulose, harsh and environmentally unfriendly chemical solvents have been used to dissolve cellulose. Most commonly in industry, the viscose or lyocell process is used [30]. The viscose process uses CS_2_ to derivatise cellulose going from alkali cellulose to cellulose xanthate. This is essential to improve the molecular rearrangement in the product formation but produces sulphur byproducts: sodium sulphate and hydrogen sulphide. An appropriate level of substitution controls solubility and kinetic hindrance [34]. The entire viscose process is a major environmental concern due to the emission of CS_2_ and H_2_S [34]. Some aqueous solvents have achieved solubility without harmful emissions or high energy consumption, such as Yue Xi et al., who utilised an aqueous AlCl_3_/ZnCl_2_ solvent system to dissolve cellulose at room temperature. It was proposed that the smaller Al^3+^ ions first penetrated to break hydrogen bonds and provide additional coordination sites. Larger Zn^2+^ ions then break more hydrogen bonds to trigger diffusion and dissolution [35,36]. These salts, however, can still impact aquatic environments [37]. Hence, it is essential to utilise improved solvent systems where possible.

Ionic liquids (ILs) are a sustainable solvent class growing in popularity. These are defined as salts that melt below 100 °C. Typically, these have a heterocyclic, non-hydrogen bonding, organic cation with asymmetry and ‘awkward’ conformations that frustrate crystallisation and reduce the T_*m*_ [37,38]. They are valued due to their high dissolving ability, negligible vapour pressure, chemical and thermal stability, non-flammability, and potential recyclability but can be toxic by various mechanisms triggering membrane rupture [30,39,40]. ILs allow for the customisation of nucleophilicity and ability to break hydrogen bonds via alterations to the anion or cation [37]. 1-Ethyl-3-methylimidazolium acetate (EmimAc) is the most commonly used cellulose solvent and can dissolve up to 27 wt% cellulose at room temperature and with low moisture content [30,37,39]. Despite these benefits, the strong anion–cation association of ILs can cause high viscosity, which can affect dissolution times and reduce effectiveness [36].

Viscosity-reducing polymer mobility can be combated with cosolvents or by increasing temperature [24]. Dimethyl sulphoxide (DMSO) acts as an efficient, available, and affordable cosolvent for this purpose [41,42,43]. DMSO is a suitable cosolvent, as it is aprotic and highly polar and hence does not impede interactions between IL anions and cellulose [42,43]. DMSO also offers environmentally friendly sustainability, as it is produced as a by-product from paper production [43]. It can be easily recycled and separated from cellulose/IL/DMSO mixtures by distillation [44]. Computational and experimental studies indicate that DMSO in these systems acts as an ‘innocent cosolvent’, which is a cosolvent that does not affect the solvation mechanism [13,43,45]. Hawkins et al. showed that DMSO addition affects the rate but not the activation energy (E_a_) of dissolution [43]. Similarly, Tomimatsu et al. showed that the solubility of cellulose in binary IL/DMSO mixtures is correlated with the hydrogen bond basicity β, and that β does not change with increasing the DMSO mole fraction (up to 0.9 DMSO mole fraction [42]). Also, the preferential association of DMSO around IL cations makes anions more available for dissolution in binary systems [46,47]. Lastly, DMSO lowers viscosity and improves mass transport in systems by reducing monomeric friction coefficients in biopolymer/IL/DMSO solutions [43,48]. Hence, the DMSO addition to IL systems can improve the total biopolymer solubility and dissolution speed.

Despite this evidence, investigations into this solvent system in dissolution conditions for application in composites are rare [6,13,43]. SF and hybrid systems also remain poorly understood despite their intriguing applications [49,50,51]. Using solvent systems that are not optimised for a specific process can impact material results. For example, longer dissolution times at higher temperatures can incur biopolymer degradation [33]. Hence, optimising the speed and effectiveness of the dissolution process offers both greater efficiency and retained material quality.

In this study, the EmimAc:DMSO solvent system is tested and optimised for the dissolution of SF fibres and MCC in conditions similar to those utilised in composite preparation studies [2,52,53] but not yet investigated with respect to their solution behaviours. The EmimAc:DMSO ratio is systematically varied for the dissolution of SF fibres and MCC to establish an optimal solvent composition for both biopolymers, which is found to differ. Then, the established optimal solutions are tested at a range of weight percentages of the biopolymer. Polarised optical microscopy is utilised to establish the presence or lack of undissolved biopolymer contents, and the dissolution behaviour is probed with rheology and nuclear magnetic resonance (NMR). Characterisation at multiple length scales allows for the understanding of macroscopic and molecular dissolution behaviours. Ultimately, the proposed systems are demonstrated to produce homogeneous solutions, which will then have application in the production of hybrid biopolymer films and composites.

## 2. Materials and Methods

### 2.1. Materials

Degummed *Bombyx mori* silk thread was purchased online (mulberry undyed spun silk from Empress Mills, Colne, UK) and stored under dry conditions. After dissolution, this silk is referred to as SF, as it comprises mostly SF. PH-101 MCC was purchased from Avicell with an approximate 50 μm particle size. Images of silk fibres and MCC can be seen in Figure S1. The IL, EmimAc, was purchased from Proionic, with a purity of 97%. All EmimAc, cellulose, and silk were dried overnight at 60 °C under vacuum before use. DMSO with a purity of 99.9% and silicone oil were purchased from Sigma-Aldrich (Darmstadt, Germany). Methanol was purchased from Fisher Scientific (Loughborough, UK), with purity of 98%.

### 2.2. Sample Preparation

MCC or silk fibres were weighed according to the biopolymer weight percentage of the sample, and solvents were weighed according to the target solvent composition. Polymeric solids were firstly dispersed in the relevant weight of DMSO, then stirred and preheated to 100 °C for 30 min. The relevant weight of EmimAc was preheated at 100 °C for 30 min then mixed with the dispersed solids in DMSO. Solutions were then stirred for 48 h, at 100 °C, at 200 rpm to produce pale yellow to dark amber transparent solutions. SF solutions showed a darker colour than cellulose solutions. All dissolution was performed in a sealed atmosphere to minimise water uptake. Throughout this study, the solvent composition is referred to as the ratio of EmimAc to DMSO in the form EmimAc:DMSO. The biopolymer content is stated as a weight percentage of the total solution, for example, “10 wt% SF”.

After preparation, all samples were stored in sealed vials at room temperature to prevent moisture uptake. The results of subsequent analyses were averaged over at least three measurements unless otherwise mentioned.

To indicate if the pretreatment of samples with DMSO affected the solution properties, separate samples were prepared without dispersal in DMSO. This was performed in the optimal solvent composition as described in Section 3.1. Imaging of these samples (see Table S1) showed slower dissolution but no difference in the ultimate solution behaviour.

### 2.3. Optical Microscopy

During dissolution, representative 1 mL samples were taken at various dissolution times up to 48 h and imaged on glass slides. Images were taken at 20× magnification using a Leica cross-polarised light microscope (London, UK) with a Nikon D7200 digital camera (Tokyo, Japan). Multiple images were taken across the whole sample to ensure that the results were fully representative of bulk sample behaviour, though they show an example of a local region.

### 2.4. Rheology

Rheological measurements were performed using an Anton Paar MCR302 stress-controlled rotational rheometer (Luton, UK) with 25 mm parallel plate geometry. The temperature was controlled with a P-PTD200/62/TG Peltier system (Luton, UK) and a circulating bath. Steady shear experiments were examined at a shear-rate range from 1 to 100 s^−1^ at 100 °C. This range was chosen to minimise the effects of DMSO evaporation, by reducing the run time of individual experiments. To minimise water uptake and DMSO evaporation during experiments, the edges of the sample were coated in a low-viscosity silicone oil, and a solvent trap loaded with DMSO was prepared around the sample. This minimised the effects of solvent evaporation and water evaporation on solution viscosity [54,55]. Each sample was heated to the desired measurement temperature for 1 min and then pre-sheared for 1 min at 1 s^−1^, to ensure adequate heating throughout.

Measurements were repeated three times, and all values and sweeps were given from averages of at least three runs. In samples with Newtonian behaviours, viscosities at 1 s^−1^ before and after testing were taken to check the effects of water uptake and evaporation. Values within the bounds of uncertainty indicated that the effect of solvent evaporation was negligible during these tests. Due to the small shear rate range tested, and Newtonian behaviours seen in the range, zero shear rate viscosities derived from a cross equation fitting were deemed inappropriate. Instead, viscosity values were taken from an average over plateaued regions without significant noise.

### 2.5. Nuclear Magnetic Resonance

^1^H NMR proton spectra were acquired using a Magritek Spinsolve desktop NMR spectrometer at 25 °C. Sixteen scans were taken with a 3.2 s acquisition time, a 4 s repetition time, and 90° pulse angle. In our analysis, spectral band ‘e’, as defined in Section 3.2.3 and corresponding to the EmimAc cation methyl group, was used as an internal reference signal and assumed to have a fixed chemical shift independent of the biopolymer concentration. Other ^1^H NMR studies on imidazolium-based ILs indicate that the chemical shift of this spectral band is largely independent of extrinsic variables, such as IL concentration in water/IL solutions and cellobiose concentration when solvated in EMIMAc [56,57,58].

## 3. Results and Discussion

### 3.1. Effects of Binary Solvent Composition

The initial solvent composition was investigated at 10 wt% biopolymer content, as this is well below the quoted saturation values of both cellulose and SF at 25 wt% and 20 wt%, respectively [56,59]. A weight percentage of 10% was commonly used in studies and seen to produce the resulting materials of high strength [60]. It is of importance to note that achieving a maximal polymer concentration in solution was a priority due to the associated material property improvements [5]. This is due to the highly associated polymer chains promoting crystallite formation and increasing interaction density improving network strength [5].

#### 3.1.1. Optical Microscopy

The birefringence of silk and cellulose biopolymers was used to ascertain the total dissolution of solutions by polarised light microscopy of the sample [51,60,61]. Polarised optical microscopy of 10 wt% solutions of cellulose in various EmimAc:DMSO ratios can be seen in Table 1, sampled at various times up to a maximum of 48 h.

Table 1 indicates that the system with a 2:8 EmimAc:DMSO solvent ratio dissolved most quickly as shown by the lack of birefringent content after two hours. This shows agreement with the studies by Ren et al. and Tomimatsu et al., who found system optima at 0.09–0.5 and 0.2 IL mole fraction for the dissolution of MCC in EmimAc:DMSO solvent systems [41,42]. As a control, no dissolution was seen in a 100% solution of DMSO (0:1) in Table 1. Next, a similar set of experiments was conducted with silk fibres. Optical microscopy of 10 wt% solutions of SF in various EmimAc:DMSO ratios can be seen in Table 2, sampled at various times up to a maximum of 48 h.

Table 2 shows that the system with 8:2 EmimAc:DMSO solvent ratio dissolved most quickly. Very interestingly, this shows a large difference from the optimal EmimAc:DMSO ratio found for MCC, which was 2:8 EmimAc:DMSO as described above. This deviation in optimal solvent composition is impacted by biopolymer choice and physical form. The hierarchical structure impacts the dissolution speed at the macroscopic level by changing bulk viscosity and aggregation behaviour [24,33,62]. At the molecular level, biopolymer chemistry can impact monomeric friction coefficients, solvent thermodynamic quality, and IL dissociation [13,15,41,42,43,45,46,47,48,56,63].

#### 3.1.2. Rheology of Samples

**Figure 1:**
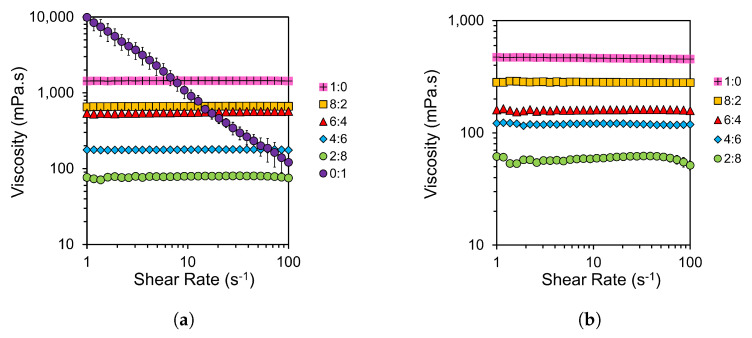
Logarithmic plots of shear rate dependence of the steady shear viscosity of (**a**) 10 wt% cellulose and (**b**) 10 wt% SF solutions at various EmimAc:DMSO ratios after dissolution for 48 h.

Rheology was performed to investigate the viscosity of solutions after 48 h, at which time the cellulose and SF was completely dissolved in most samples. These tests were performed between 1 and 100 s^−1^, and at the same temperature as the dissolution performed in similar composite preparation studies of 100 °C [6,53,59,64]. Though total dissolution was the primary concern of this study, a secondary priority was to reduce solution viscosity to ease sample preparation for any future planned work on the manufacture of hybrid biocomposites. Reduced viscosity increases matrix penetration into supporting fibres for use in reinforced composites, though it must also be considered that too low a viscosity can cause excess material loss during preparation [6]. The shear rate sweeps of both cellulose and SF at different solvent compositions can be seen in Figure 1.

**Figure 2:**
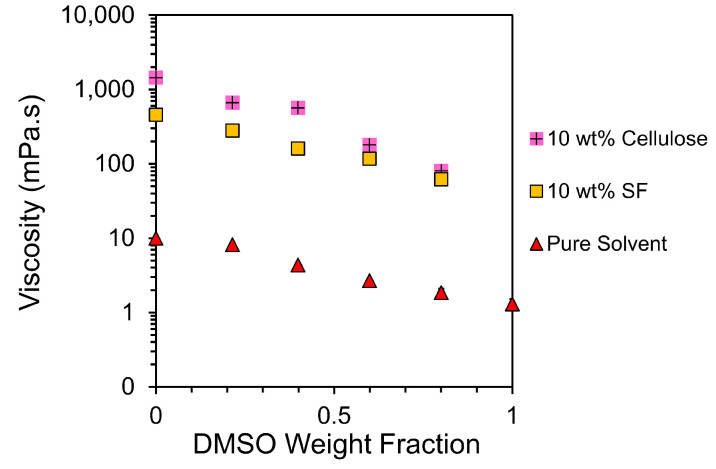
Plot of logarithmic viscosities against the weight fraction of DMSO in solvent. The DMSO/SF solution was unable to be tested, and the DMSO/MCC solution showed significant deviation from Newtonian behaviour, so both were excluded.

At the chosen temperature of 100 °C, Newtonian behaviour is noted across most of the shear rate sweeps shown in Figure 1a,b. Deviations from Newtonian behaviour are seen at 0:1 EmimAc:DMSO for 10 wt% cellulose solutions and at 2:8 EmimAc:DMSO for 10 wt% SF solutions. This is supporting evidence for the optical micrographs shown in Figure 1 and Figure 2, where these were the only two sampled solutions at 48 h that showed remaining undissolved content. SF fibres in a 0:1 EmimAc:DMSO could not be rheologically tested due to significant jamming from undissolved fibres. Similarly to Figure 1, Owens et al. found that increasing the solution temperature of cellulose in EmimAc reduces viscosity and increases the shear rate at which shear thinning behaviour is noted [24]. The intrinsic viscosity was also reported to decrease with elevated temperature due to a decrease in solvent quality and polymer chain size [33]. Conversely, in studies at lower temperatures or without a DMSO cosolvent, shear thinning was commonly observed [24,32,33,48,49,50].

Average solution viscosities in terms of EmimAc:DMSO ratios and the biopolymer type can be seen in Figure 2, highlighting the exponential decrease in viscosity seen with the addition of DMSO.

Figure 2 indicates three main aspects. First, the average viscosities of the 10% weight solutions for both cellulose and silk are two orders of magnitude higher than the equivalent pure solvents at the same EmimAc:DMSO ratio. Second, as expected, the average viscosity of the solutions falls as the DMSO content is increased. And thirdly, the average viscosities of the two biopolymer solutions are comparable for all EmimAc:DMSO ratios.

**Figure 3:**
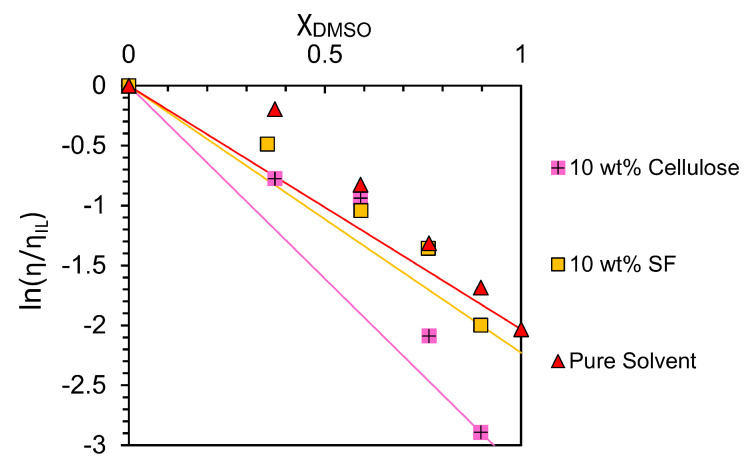
Logarithmic plot of viscosities against the DMSO mole fraction with linear fittings calculated according to Equation (1). The DMSO/SF solution was unable to be tested, and the DMSO/MCC solution showed significant deviation from Newtonian behaviour, so both were excluded from the fittings.

The effect of organic cosolvents on the viscosity of ILs has previously been expressed by an exponential equation [46,48]. This relationship between the viscosity of the IL/cosolvent mixture and the concentration of the cosolvent can be described by the following equation [46,48]:

(1) $$\ln\eta =\ln\eta_{IL}-\frac{x_{DMSO}}{\alpha }$$

where η and $\eta_{IL}$ are the viscosities of a given solution and the solution with a pure EmimAc solvent; $x_{DMSO}$ is the mole fraction of DMSO in the solvent mixture; and α is a constant. When $x_{DMSO}=1$, Equation (1) can be rewritten as the ratio of viscosities of solutions of pure EmimAc and pure DMSO solutions. Therefore, this fitting tells us about the ratio of solution viscosities in the conditions shown. To best represent the logarithmic fitting behaviour modelled, the fit was performed between zero and the data point with the highest DMSO content. The fittings for solvent mixture, 10 wt% SF, and 10 wt% cellulose solutions at 100 °C are shown in Figure 3. The shear rate sweeps for pure solvent values plotted can be seen in Figure S2.

The logarithmic fitting in Figure 3 shows that pure solvent solutions at this temperature vary less with DMSO than solutions with biopolymer content. All fitting values can be seen in Table 3 below.

Both Figure 3, and the **R**_2_ values in Table 3 show deviation from the logarithmic fitting. It is reported that DMSO disrupts the dynamic ion clusters within ILs [33,46], and the small increase in experimental viscosity above the theoretical mixing law indicates weak interactions between the DMSO and IL system components [46]. The larger deviation shown in 10 wt% cellulose samples could indicate a larger effect on viscosity from DMSO/IL interactions in these solutions. Interestingly, it has also been shown that cellulose dissolution is dependent on ion mobility and IL hydrogen bond basicity β, conferring the importance of IL and DMSO interactions [42].

Based on the speed of dissolution shown in Table 1, and the Newtonian behaviours shown in Figure 1a, a solvent composition of 2:8 EmimAc:DMSO will be further investigated for the effective dissolution of cellulose. This is a similar composition to the optima proposed by Ren et al. and Tomimatsu et al. for the rapid dissolution of MCC but differs from studies on the dissolution of cellulose fibres [6,41,42,43].

Based on Table 2 and Figure 1b, a solvent composition of 8:2 EmimAc:DMSO will be further investigated for the dissolution of SF. Though this composition choice may be influenced by the biopolymer type, Seoud et al. found system optima between 0.5 and 0.9 DMSO mole fraction in similar IL/DMSO binary solvent mixtures [51]. This implies the largest effect, dictating that optimal IL/DMSO compositions may be of a biopolymer form or macroscopic hierarchical structure.

While studying flax fibre dissolution, Hawkins et al. reported a reduction in the dissolution rate above 50 wt% DMSO [43]. This was attributed to a change in DMSO preferential association from cation to anion above 0.6 mole fraction DMSO [46,47,66], despite an activation energy of dissolution of 100 ± 10 kJ mol^−1^ independent of the DMSO concentration [43]. Conversely, lower DP cellulose forms have shown effective dissolution at higher DMSO concentrations [41,42]. This contradiction implies that IL/DMSO systems are effective solvents at high DMSO concentrations but are unable to disrupt larger, more entangled biopolymer networks. This could indicate that longer chain biopolymers (like MCC compared to fibres [5,33]) are governed primarily by macroscopic viscosity and diffusive effects in these systems [5,35,42,67]. This supports Liang et al., who found a difference in dissolution speed across three different arrangements of cotton fibre bundles, despite a consistent activation energy of dissolution [62]. Most studies have focused on dissolution mechanisms at the mesomolecular level as dictated by cellulose’s largely insoluble amphiphilic structure [40], but the work by Cuissinat and Navard highlights five modes of interaction for the dissolution of cellulose fibres in ionic liquids observable by optical microscopy [62,68]. We can therefore rationalise the different dissolution speeds with corresponding modes [68]: (1) fast dissolution by disintegration into fragments; (2) large swelling by disintegration and complete dissolution; (3) large swelling by ballooning and no complete dissolution; (4) homogeneous swelling and no dissolution; and (5) no swelling and no dissolution.

Interestingly, this discrepancy may be exacerbated by differences in the definition of dissolution across the literature. In some studies, dissolved sections are measured by total coagulated content around a partially dissolved fibre after partial solvation in an IL and coagulation in an antisolvent [6,43,62,69]. This implies that the coagulated content does not disperse fully into the solution to achieve a full solvation cage. The term ‘dissolution’ in this case may more accurately refer to the entry of solvent ions into the polymer network and the disruption of the crystalline content. Other techniques, such as small-angle X-ray scattering and NMR, can indicate dissolution to the molecular level up to high biopolymer concentrations [56,70,71,72]. In other studies, the saturation concentration is tested by the timed addition of undissolved content [41]. This could impart a greater impact from dissolution speed as opposed to truly finding a system’s saturation concentration.

### 3.2. Weight Percentage of Biopolymers in Optimal Solvent Systems

After establishing optimal EmimAc:DMSO solvent ratios for both biopolymers in a 10 wt% composition, we then investigated the total solubility of the given biopolymers in these solutions between 0 and 20 wt% biopolymer. This was to confirm the solvation behaviour over the concentration range chosen, and to establish the saturation concentration of the solvent systems used. Again, all solutions were stirred at 200 rpm, and 100 °C for 48 h to dissolve prior to testing.

#### 3.2.1. Optical Microscopy

The optical microscopy of different weight percentage solutions of MCC in a solvent composition of 2:8 EmimAc:DMSO can be seen in Table 4.

Table 4 shows that above 10 wt% cellulose for our dissolution conditions undissolved particles at a μm length scale remain in solution. This value indicates a saturation concentration below some literature examples with lower DMSO concentrations [32,41,56]. This could be linked to the lower total quantity of IL ions stabilising the solvation shell in solution but only confirms the macroscopic behaviours [24]. Molecular behaviours with changes in weight percentage are discussed in Section 3.2.3. The optical microscopy of different weight percentage solutions of SF in a solvent composition of 8:2 EmimAc:DMSO can be seen in Table 5.

Table 5 shows SF solutions with undissolved fibres above 11 wt%. This implies a saturation concentration between 11 and 15 wt% in this solvent system. This is above the concentrations used in many literature examples [49,50,51], but lower than the maximum of 20 wt% SF achieved by Zhang et al. in pure EmimAc [59]. This reduced saturation concentration could indicate that the total solubility in this solvent composition is reduced by the introduction of DMSO into the solvent composition.

#### 3.2.2. Rheology

**Figure 4:**
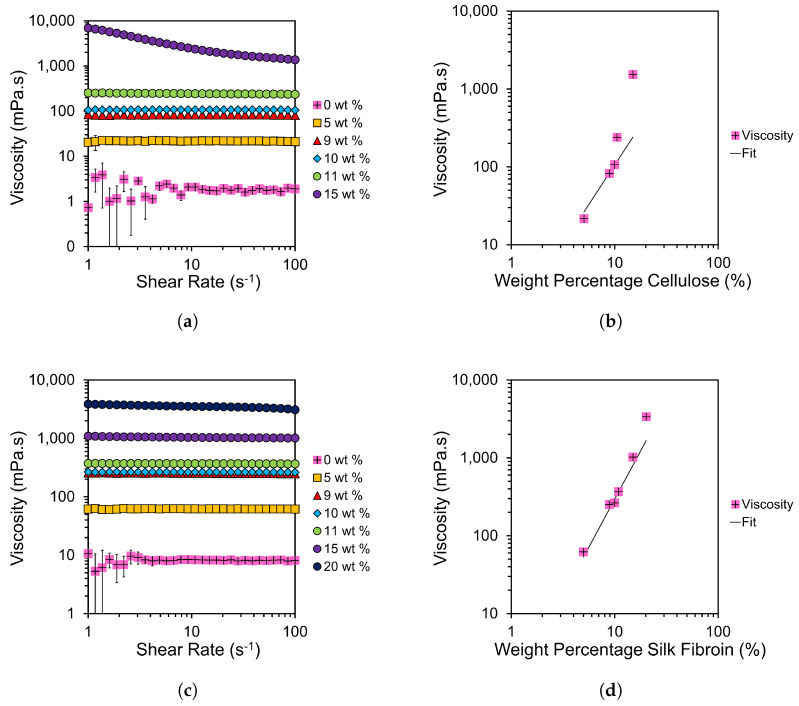
Logarithmic plots of viscosity as a function of (**a**,**c**) shear rate at each given weight percentage of cellulose and SF; and (**b**,**d**) the weight percentage of cellulose or SF with fits extrapolated according to Equation (2).

Shear rate sweeps were taken at various weight percentages of MCC in a solvent composition of 2:8 EmimAc:DMSO; see Figure 4a. Viscosities were then plotted against cellulose weight percentage; see Figure 4b. Equivalent plots for SF fibres dissolved in 8:2 EmimAc:DMSO solutions can be seen in Figure 4c,d. The viscosity values in the semi-dilute entangled regime were fitted with a power law dependency as described by Lefroy et al. [33]:

(2) $$\eta =kc^{n}$$

where η is the sample viscosity, *k* is a constant, *c* is the biopolymer weight percentage, and *n* is the power law exponent.

Figure 4a,c and Figure S2 highlight the low shear regime (of increased variation) shown in EmimAc:DMSO solutions at 0 wt% cellulose or SF. This is likely caused by the hydrogen bonding of EmimAc ions causing weak cluster networks [33]. The presence of this behaviour despite preshear indicates dynamic networks with rapid formation at low shear rates. Lefroy et al. found that this behaviour disappears with cellulose introduction due to the disruption of IL clusters by fully dissolved polymer chains [33]. We confirm this finding for both biopolymer examples.

Figure 4a,c show predominantly Newtonian behaviours below the saturation concentrations proposed in Section 3.2.1. The lack of shear thinning differs from similar weight percentage cellulose solutions in the literature at lower DMSO solvent contents and lower temperatures [32,48]. This shows an increase in the onset value of shear thinning in the samples tested at higher temperatures [24,33,48,49]. Viscosity is also reduced by the introduction of DMSO and increased temperature [24,32,33,48,49].

Viscosities seen for SF solutions with 8:2 EmimAc:DMSO solvent ratios, in Figure 4c, are higher than the equivalent weight percentages in cellulose solutions with 2:8 EmimAc:DMSO solvent composition. This shows the DMSO content of the solvent system to be more impactful than the biopolymer in controlling solution viscosity in this case. Despite this, SF solutions are noted to be less viscous than equivalent cellulose solutions due to greater chain flexibility and/or lower molecular weights [5,7,64].

By fitting with Equation (2) for values up to 10 wt% cellulose in Figure 4b, and up to 11 wt% SF in Figure 4d, a transition from the semi-dilute entangled regime to incomplete dissolution is shown. This deviation supports the optical micrographs in Table 4 and Table 5 in showing showing a saturation concentration at 10 wt% and 11 wt% for cellulose and SF respectively. All values tested are above the literature entanglement concentrations for MCC C_e_ = 1.3±0.1 [48,73], so they show only the semi-dilute entangled regime. Our fittings show an exponent of *n* = 2.3 ± 0.1 for cellulose solutions and *n* = 2.2 ± 0.1 for SF solutions, which match the results by Gericke et al. for the equivalent region of pure EmimAc/MCC solutions at 100 °C [32].

Predictions for neutral polymers give a power law index, *n*, of 1, 2 and 14/3 in a θ solvent, and 1, 1.3 and 3.9 in a good solvent for neutral polymer solutions in dilute, semi-dilute unentangled, and semi-dilute entangled regimes, respectively [73]. *n* values for both SF and cellulose solutions are less than the theoretical predictions for θ solvents. This indicates a deviation from θ-solvent behaviour with increased temperature, as previously shown with similar IL-biopolymer solutions, but that the solvent composition tested does not effect the solvent quality [32,48,63,73].

Despite variations in the DMSO concentration, the entanglement state of the SF or cellulose biopolymer in EmimAc:DMSO solutions tested is relatively unchanged. This finding extends from the work by Lv et al., who showed that an increased DMSO concentration (50 wt%) does not affect the entanglement state but reduces the monomeric friction coefficient and hence the viscosity and relaxation times [48]. Tomimatsu et al. also confirmed a maximum cellulose solubility at approximately 0.8 DMSO mole fraction due to an increase in ion mobility [42]. They showed that the IL solvation ability correlates with hydrogen bond basicity β, and that the β of EmimAc is largely constant up 0.9 DMSO mole fraction [42]. This supports our conclusion that the solvent quality is maintained from 1:0 to 2:8 EmimAc:DMSO solvent compositions.

#### 3.2.3. NMR

**Figure 5:**
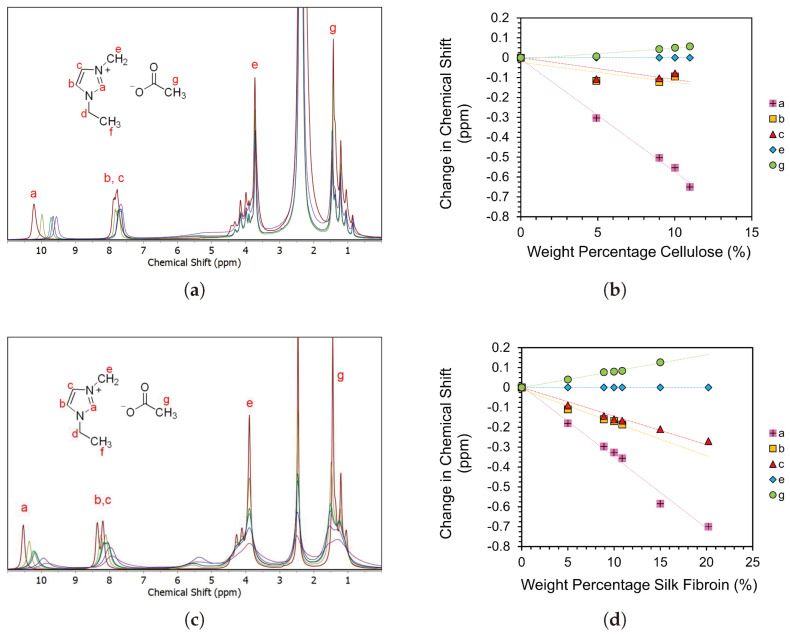
(**a**,**c**) ^1^H NMR spectra of 2:8 EmimAc:DMSO solutions at various cellulose concentrations and 8:2 EmimAc:DMSO solutions at various SF concentrations, respectively. The inset shows the chemical structure of the [Emim]^+^ and [Ac]^−^ ions of EmimAc. Peaks signals labeled a–g are attributed to equivalent proton environments seen on the EmimAc diagrams. (**b**,**d**) show the concentration dependence of the change in ^1^H NMR chemical shifts for the proton environments labelled in the inset molecular diagram. Linear fits are included as a guide to the eye. The error bars are approximately equal to the size of the data points used. Samples above 11 wt% MCC are too viscous to be prepared in the given NMR tubes. In SF fibre solutions with undissolved content, the solution is pipetted away from the undissolved content for NMR analysis. All peak integrals and tabulated raw data can be seen in Tables S2 and S3.

To investigate the molecular behaviour of solutions, NMR is performed. Cellulose peaks cannot be clearly defined due to the relatively low population of protons in the studied ranges [56], and hence the local interactions and molecular behaviours are studied by the chemical shift dependence of EmimAc peaks on the biopolymer concentration as shown in Figure 5.

Figure 5a,b show a shift in the EmimAc chemical environment up to 11 wt% MCC, indicating that the local environment for EmimAc cations is changing [56]. This is consistent with the coordination of IL ions with cellulose hydroxyl groups as proposed by Zhang et al. for the dissolution and solvation mechanism of cellobiose in EmimAc [56,58]. The change in chemical shift seen implies an increase in the cellulose content in solution and hence confirms alterations in the molecular level behaviour of these samples.

A shift in spectral bands is also seen with increasing the SF content in Figure 5c,d, indicating the association of IL cations with SF hydroxyl groups equivalent to that seen in cellulose and cellulose-derived biopolymers [56,58,69]. This confirms the SF dissolution mechanism theorised by Phillips et al. that IL ions disrupt the hydrogen bonding domains in β-sheet/crystalline regions of SF, incurring solvation [5,69].

Figure 5d shows the continued chemical shift at concentrations in which fibres were seen to be undissolved (15–20 wt% SF). This highlights the distinction between macroscopic and microscopic dissolution behaviours [33,62,74]. Though the solution increased in concentration of SF molecules, the solvent system was not capable of dissolving the fibres as seen in Section 3.2.1. The difference between local molecular (NMR) and bulk (rheology and optical microscopy) saturation concentrations indicates a distinction between the solvent thermodynamic quality at the molecular level, and the ability of a solvent system to disrupt and disperse polymer networks.

Notably, peaks in Figure 5c are more clearly distinguished than those in Figure 5a due to the larger relative population of EmimAc protons in the solvent composition. Samples with higher weight percentages of biopolymers are also higher in viscosity, which reduce the sample isotropy and hence signal quality [56]. Any data points omitted in Figure 5b,d are excluded due to the peaks being poorly distinguished at higher biopolymer concentrations.

### 3.3. Preparation of Hybrid Solutions and Coagulated Films

Having achieved the dissolution of both SF and cellulose biopolymers and created solutions of minimal viscosities, solutions of 10 wt% cellulose in 2:8 EmimAc:DMSO solvent ratio and 10 wt% SF in 8:2 EmimAc:DMSO solvent ratio are prepared by with the method described in Section 2. Equal masses of these solutions are mixed at 200 rpm for 30 min at 100 °C to indicate the effectiveness in producing hybrid solutions for composite preparation. This causes no coagulation or aggregation out of solution as indicated by the optical microscopy in Figure S3 and the Newtonian behaviour with a viscosity of 142 ± 2 mPa.s as seen in Figure S4. This implies the homogeneity and stability of the biopolymers in solution and indicates the effectiveness of the dissolution method for future hybrid biocomposite preparation.

**Figure 6:**
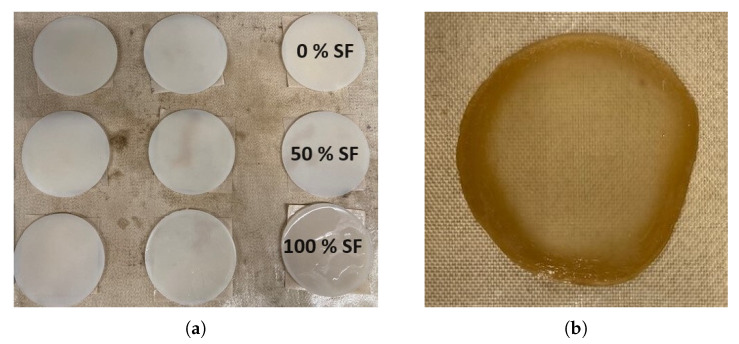
Example photos of (**a**) wet gels with weight percentage of silk fibroin from 0–100 wt% and (**b**) an example dried film of 15 wt% SF content.

A film preparation method is then developed from the literature examples [53]. The polymer solution is poured into a circular petri dish (10 cm diameter) and left to de-aerate for 2 h at 70 °C under vacuum. The cast film is then coagulated in a methanol atmosphere statically for 24 h, by placing under a vacuum environment with 200 mL of methanol. The film is then washed in deionised water (5 L) for 48 h. The water is replaced twice in that period. This wet gel is then dried at room temperature and humidity for 6 h. Films are then pressed between flat metal sheets (≈30 N using bulldog clips) to minimise warping due to differential shrinkage during drying and cooling, then dried for 24 h at 60 °C [53]. By altering the weight percentage ratio of the mixed solutions, films are successfully produced of 0–100 wt% SF content as shown in Figure 6. Having developed the optimal EmimAc:DMSO ratios for dissolving the two biopolymers, and blended them, a subsequent publication will examine the mechanical properties of a range of hybrid biopolymer films with differing cellulose/silk ratios.

## 4. Conclusions

The effect of the EmimAc:DMSO solvent ratio was first investigated for the dissolution of a 10 wt% fraction of SF and MCC at 100 °C. MCC was most quickly and efficiently dissolved in a 2:8 EmimAc:DMSO solvent mixture, and SF fibres were most efficiently dissolved in an 8:2 EmimAc:DMSO solvent mixture. The steady-shear rheology of solutions at various solvent compositions with 10 wt% biopolymer content (for samples dissolved for 48 h) was measured and showed mostly Newtonian behaviours. Deviation from Newtonian behaviour was only found for samples that showed undissolved material in optical microscopy, offering an additional method to establish when full dissolution had occurred. The steady shear viscosities were then plotted as function of the DMSO mole fraction, and deviation from the exponential mixing rules of solvents indicated that EmimAc:DMSO interactions affected the viscosity significantly in all solvent systems studied. This showed the impact of solvent–cosolvent interactions on bulk viscosity. Comparison with trends in the literature indicated that the dissolution behaviour of different solvent compositions was greatly affected by the biopolymer form [41,42,43,62]. Larger hierarchical structures or longer-chain biopolymer networks required solutions with a higher IL concentration to effectively disrupt the polymer network.

The total solvation ability of the selected optimal solvent mixtures was then tested at different biopolymer weight percentages. The macroscopic dissolution behaviours to the μm length scale were investigated with optical microscopy and rheology. Imaging showed undissolved content at 11 wt% cellulose upwards and from 15 wt% SF upwards. This implied a saturation concentration between 10–11 wt% and 11–15 wt% for cellulose and SF respectively. This was confirmed by deviation from a power law dependency in viscosity against sample concentration at these values. This fitting in the semi-dilute entangled region showed agreement with similar studies at lower DMSO contents, with power law exponents, *n*, of 2.2 and 2.3 ± 0.1 for SF and cellulose solutions, respectively [32]. This confirmed that the solvent systems thermodynamic quality and biopolymer conformations remained relatively constant from 1:0 to 2:8 EmimAc:DMSO solvent compositions.

Having established that effective solvation conditions hybrid solutions were prepared and then used to create blend hybrid biopolymeric materials, future investigation will build on this finding and characterise biopolymer films as well as reinforced hybrid biocomposite examples.

Lastly, a discrepancy between the molecular and macroscopic behaviours is evidenced by the difference in the apparent saturation content in the SF fibres in this study. NMR evidence shows continued solvation up to 20 wt% SF as expected from the literature [59], beyond the optically observed saturation point between 11 and 15 wt% SF. Hence, the thermodynamic quality of the solvent at the molecular level is shown to be beyond the macroscopic dissolution achievable with this procedure. This highlights the necessity of investigating multiple length scales in evaluating solvation behaviour.

In future studies, computational coarse-grained modelling may be able to bridge the understanding between macroscopic, mesoscopic, and microscopic dissolution behaviour in hybrids systems [15,75]. This could help elucidate further the effect of macroscopic arrangement, hierarchical structure, biopolymer sources, and DP on complex solution behaviours [62]. In particular, SF specific studies on this topic would add to the current understanding greatly. Investigation into composites manufactured with this understanding could help greatly in fields of biomedical and materials research [4,76,77,78,79]. Further research of ionogel devices created with the solutions studied here could aid the development of next-generation sustainable electrochemical devices [13,80]. For example, ionic liquids can act as high-performance electrolytes in cellulose-based flexible super-capacitors [80]. It is important to note that this study lacks the direct observation of dissolution mechanisms occurring in the solvent system. This could be improved by the use of cryogenic transmission electron microscopy imaging in future studies [72]. A more precise optimum could also be derived for this system with more solvent/cosolvent ratio variations, through a design-of-experiments approach.

This research improves the understanding of biopolymer dissolution in EmimAc:DMSO systems and shows how this could be applied to produce hybrid biopolymer materials. It provides further insights into the dissolution behaviours occurring on a macroscopic and local level, which is crucial to understand the properties of materials coagulated from IL solutions. The knowledge contributed will help to implement and hasten further developments in the field of sustainable materials.

## Figures and Tables

**Table 1:** Table showing the dissolution behaviour of Avicell MCC over time (up to 48 h) at different EmimAc:DMSO solvent ratios. All images are taken at ×20 magnification using transmission cross-polarised light microscopy. Scale bars shown are equivalent to 1 mm. Table heading E:D refers to the solvent ratio between EmimAc and DMSO.

E:D	1 h	2 h	4 h	24 h	48 h
1:0	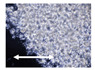	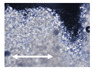	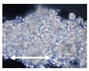	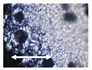	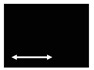
8:2	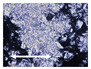	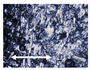	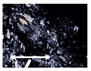	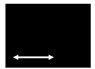	
6:4	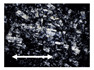	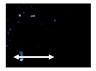	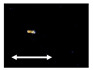	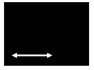	
4:6	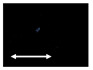	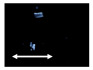	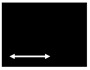		
2:8	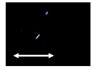	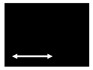			
0:1	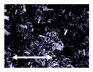	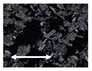	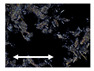	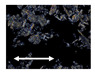	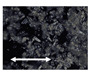

**Table 2:** Table showing the dissolution behaviour of SF over time (up to 48 h) at different EmimAc:DMSO solvent ratios. All images are taken at ×20 magnification using transmission cross-polarised light microscopy. Scale bars shown are equivalent to 1 mm. Note the presence of undissolved solid after 48 h at 2:8 EmimAc:DMSO solvent composition. Table heading E:D refers to the solvent ratio between EmimAc and DMSO.

E:D	1 h	2 h	4 h	24 h	48 h
1:0	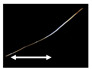	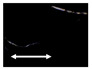	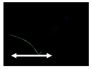	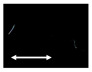	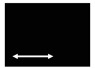
8:2	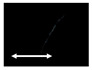	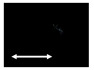	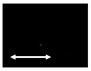	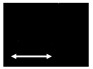	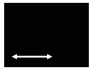
6:4	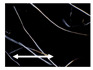	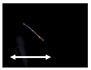	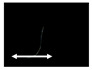	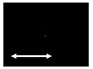	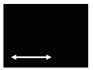
4:6	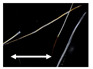	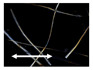	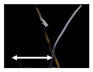	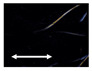	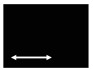
2:8	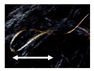	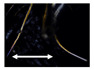	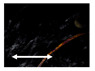	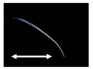	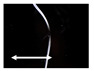
0:1	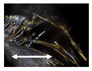	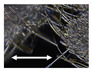	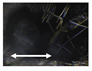	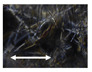	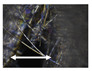

**Table 3:** Table of α and **R**^2^ values for log fitting of IL/DMSO solutions with Equation (1). Values for 25 °C pure solvent fitting are taken from the literature [48]. Errors in α values are estimated from the least-squares fitting using the ‘jack-knife’ or numerical substitution method [65].

Biopolymer Solute	Temperature/°C	α	R^2^
10 wt% Cellulose	100	0.31 ± 0.01	0.88
10 wt% SF	100	0.44 ± 0.01	0.95
None	100	0.49 ± 0.01	0.91
None	25	0.15	0.99

**Table 4:** Table showing the dissolution behaviour of MCC at various weight percentages after 48 h of dissolution with stirring at 100 °C. All images were taken at ×20 magnification using transmission polarised light microscopy. Scale bars shown are equivalent to 1 mm. Note: at 11 weight percent of cellulose in solution, undissolved crystalline content can be seen on the μm length scale as highlighted.

Weight Percentage of Cellulose in Solution			
0 wt%	5 wt%	9 wt%	10 wt%
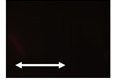	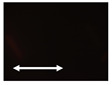	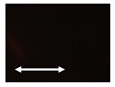	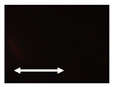
11 wt%	15 wt%	20 wt%	
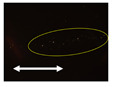	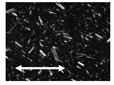	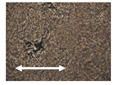	

**Table 5:** Table showing the dissolution behaviour of SF fibres at various weight percentages after 48 h of dissolution with agitation at 100 °C. All images were taken at ×20 magnification using transmission polarised light microscopy. The scale bars shown are equivalent to 1 mm.

Weight Percentage of Silk Fibroin in Solution			
0 wt%	5 wt%	9 wt%	10 wt%
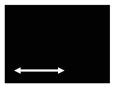	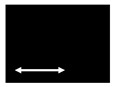	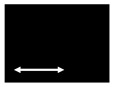	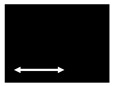
11 wt%	15 wt%	20 wt%	
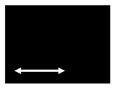	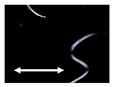	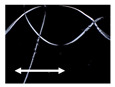	

## Data Availability

All data needed to evaluate the conclusions in the paper are present in the paper and/or the Supplementary Materials. In addition, the data associated with this paper are openly available from the University of Leeds Data Repository at https://doi.org/10.5518/1583.

## Supplementary Materials

The following supporting information can be downloaded at: https://www.mdpi.com/article/10.3390/ma17215262/s1, Figure S1: Polarized light microscopy image of Avicel Ph-101 microcrystalline cellulose and degummed silk fibres; Figure S2: Logarithmic plots of shear viscosity against shear rate in EmimAc/DMSO mixtures; Figure S3: Polarized light microscopy image of 10 wt% hybrid biopolymer solution; Figure S4: Logarithmic plots of shear viscosity against shear rate of a 10 wt% hybrid biopolymer solution; Table S1: Polarized light microscopy image of 10 wt% Avicell microcrystalline cellulose in 2:8 EmimAc:DMSO solvent composition without initial dispersion; Table S2: Raw data from ^1^H NMR spectra of 8:2 EmimAc:DMSO solutions at various SF concentrations; Table S3: Raw data from ^1^H NMR spectra of 2:8 EmimAc:DMSO solutions at various cellulose concentration.

## Abbreviations

The following abbreviations are used in this manuscript:

SF	Silk Fibroin
MCC	Micro-Crystalline Cellulose
DP	Degree of polymerisation
IL	Ionic Liquid
EmimAc	1-ethyl-3-methylimidazolium acetate
DMSO	Dimethyl Sulphoxide
NMR	Nuclear Magnetic Resonance
