# Supplementary material for: Understanding the Dissolution of Cellulose and Silk Fibroin in 1-ethyl-3-methylimidazolium Acetate and Dimethyl Sulphoxide for Application in Hybrid Films
Source: Materials (Basel). 2024 Oct 29;17(21):5262. doi: 10.3390/ma17215262 (PMC11547638; doi:10.3390/ma17215262)
Supplement: Supplementary file 1 [file materials-17-05262-s001.zip › Supplementary_Figures.pdf]

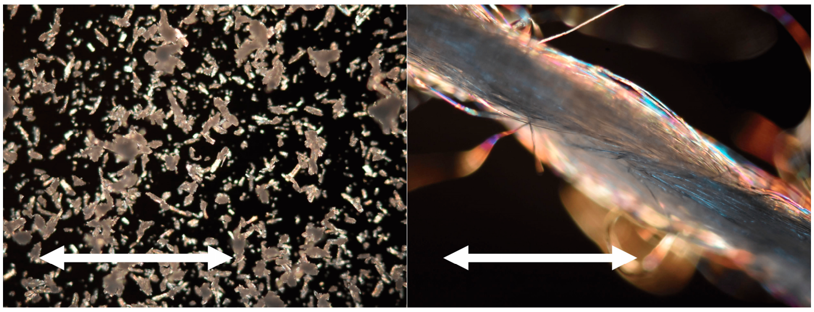

Supplementary Figure S1: Images of Avicell Ph-101 microcrystalline cellulose, and degummed *Bombyx Mori* silk fibre. All images are taken at x20 magnification using transmission cross-polarised light microscopy. Scale bars shown are equivalent to 1 mm.

Time Elapsed			
1 hr	3 hr	6 hr	24 hr
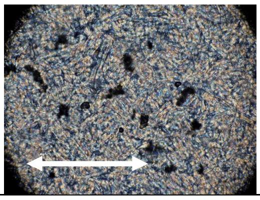	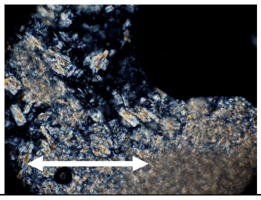	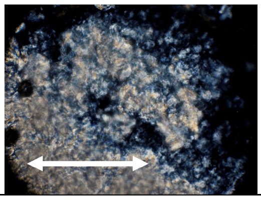	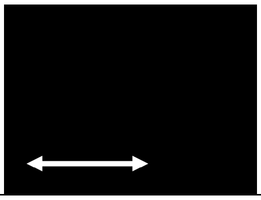

Supplementary Table S1: Transmission cross-polarized optical microscopy of 10 wt% Avicell MCC solution in 2:8 EmimAc:DMSO solvent composition without initial dispersion at 100 °C. All images are taken at x20 magnification using transmission polarised light microscopy. Scale bars shown are equivalent to 1 mm.

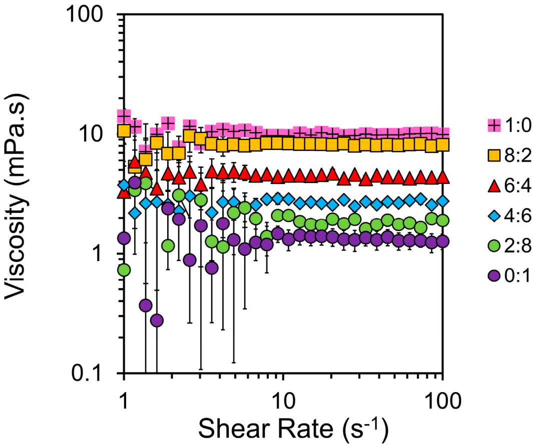

Supplementary Figure S2: Logarithmic plots of shear rate dependence of the steady shear viscosity of pure solvent solutions at various weight percentages of DMSO in the solvent composition. The legend refers to the weight ratio of EmimAc:DMSO in solution.

	Peak Label									
Weight Percentage of SF / %	a		b		c		e		g	
	Average Peak Position / ppm	Average Peak Integral	Average Peak Position / ppm	Average Peak Integral	Average Peak Position / ppm	Average Peak Integral	Average Peak Position / ppm	Average Peak Integral	Average Peak Position / ppm	Average Peak Integral
0.00	10.53	1172.97	8.36	682.22	8.19	823.18	3.89	3584.61	1.44	3451.77
4.99	10.19	1077.21	8.09	1131.77	7.94	1047.52	3.73	3087.11	1.32	3309.24
8.89	10.09	1135.95	8.05	1121.26	7.9	951.6	3.74	3167.98	1.37	3319.18
9.99	10.07	1125.56	8.05	1110.25	7.89	991.31	3.75	3179.3	1.38	3295.93
10.88	10.05	1087.48	8.05	1127.87	7.9	992.97	3.77	3205.94	1.4	3304.19
15.00	9.76	1154.31			7.79	1471.21	3.7	3581.78	1.38	3113.76
20.21	9.50	735.58			7.59	1697.97	3.56	5351.98	0.98	7310.55

Supplementary Table S2: Raw data from ^{1}H NMR spectra of 8:2 EmimAc:DMSO solutions at various SF concentrations. All data shown is prior to normalisation and correction for drift by use of peak ***e*** as a stationary reference signal.

	Peak Label									
Weight Percentage of cellulose / %	a		b		c		e		g	
	Average Peak Position / ppm	Average Peak Integral	Average Peak Position / ppm	Average Peak Integral	Average Peak Position / ppm	Average Peak Integral	Average Peak Position / ppm	Average Peak Integral	Average Peak Position / ppm	Average Peak Integral
0.00	10.23	287.67	7.86	96.04	7.78	170.82	3.73	1124.78	1.43	573.99
4.91	10.05	292.19	7.87	66.74	7.80	184.61	3.86	834.46	1.56	871.11
8.99	9.78	259.79	7.79	91.62	7.73	211.49	3.78	761.96	1.52	777.95
10.02	9.78	273.24	7.87	129.46	7.81	129.36	3.84	705.17	1.58	784.23
10.98	9.67	254.53					3.82	683.51	1.57	692.92

Supplementary Table S3: Raw data from ^{1}H NMR spectra of 2:8 EmimAc:DMSO solutions at various cellulose concentrations. Samples above 11 wt % MCC were too viscous to be prepared in the given NMR tubes. All data shown is prior to normalisation and correction for drift by use of peak ***e*** as a stationary reference signal.

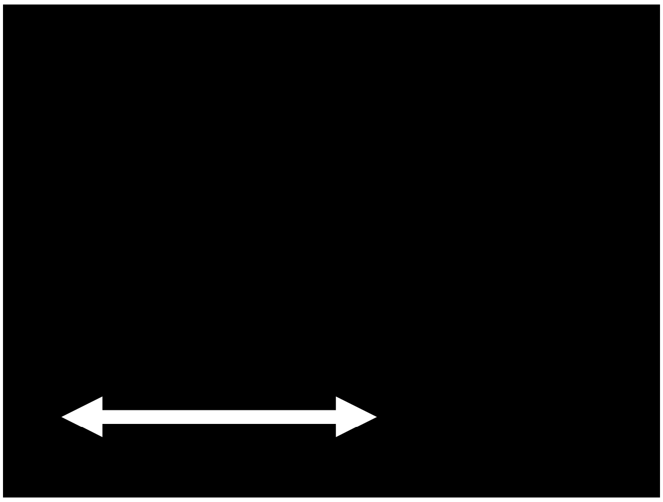

Supplementary Figure S3: Polarised optical microscopy images at x20 magnification of 10 wt % hybrid biopolymer solution. This is in a 1:1 silk fibroin:cellulose weight ratio. All images are taken at x20 magnification using transmission cross-polarised light microscopy. Scale bars shown are equivalent to 1 mm.

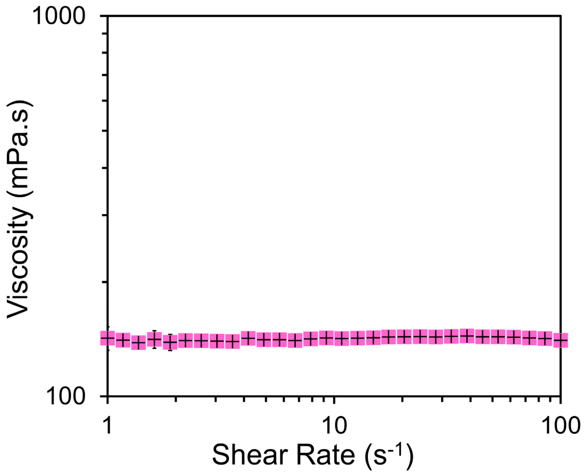

Supplementary Figure S4: Logarithmic plot of the shear rate dependence of the steady shear viscosity of 10 wt % hybrid biopolymer solution. This is in a 1:1 silk fibroin:cellulose weight ratio.
